# Characterization of ultrathin InSb nanocrystals film deposited on SiO_2_/Si substrate

**DOI:** 10.1186/1556-276X-6-601

**Published:** 2011-11-23

**Authors:** Dengyue Li, Hongtao Li, Hehui Sun, Liancheng Zhao

**Affiliations:** 1 Department of Information Materials Science and Technology, Harbin Institute of Technology, Harbin 150001, P. R. China

## Abstract

Recently, solid-phase recrystallization of ultrathin indium antimonide nanocrystals (InSb NCs (films grown on SiO_2_/Si substrate is very attractive, because of the rapid development of thermal annealing technique. In this study, the recrystallization behavior of 35 nm indium antimonide film was studied. Through X-ray diffraction (XRD) analysis, it is demonstrated that the InSb film is composed of nanocrystals after high temperature rapid thermal annealing. Scanning electron microscopy shows that the film has a smooth surface and is composed of tightly packed spherical grains, the average grain size is about 12.3 nm according to XRD results. The optical bandgap of the InSb NCs film analyzed by Fourier Transform infrared spectroscopy measurement is around 0.26 eV. According to the current-voltage characteristics of the InSb NCs/SiO_2_/p-Si heterojunction, the film has the rectifying behavior and the turn-on voltage value is near 1 V.

## Introduction

Moore's law has predicted that the number of transistors, which are placed inexpensively on an integrated circuit, doubles approximately every 2 years. This trend is expected to be maintained until at least 2020. Nowadays, 65-nm technology node of the most advanced microprocessors in production has transistor gate lengths of 35 nm, which is looking forward to be 22 nm in 2016 [1,2]. However, the inherent scaling limitations of electron devices bring many technical challenges from traditional work mode, i.e., material and basic device physics; therefore, researchers should promptly overcome or improve some of these challenges with various research breakthroughs. Ultrathin III-V compound semiconductor film growth on Si-based substrate has attracted significant attention because of the combination of the high mobility of III-V semiconductors and the well established, low-cost processing of Si technology. Paralleling with silicon-on-insulator technology, XOI was used to represent compound semiconductor-on-insulator platform [3-6]. Semiconductor NCs provide size-tunable optical and electrical properties, based on quantum confinement of charge carriers within them, high surface-to-volume ratios, and other attributes that have led to the development of a new generation of materials and devices [7-10]. Because of their various unique properties, there has been considerable interest in the synthesis of high-quality III-V compound NCs [11,12]. InSb has the smallest effective mass, the narrowest bandgap, and the highest intrinsic electron mobility of III-V semiconductors.

In this article, InSb NCs film is grown on SiO_2_/Si (111) substrate using solid-phase recrystallization by rapid thermal annealing. The remainder of this article is organized as follows. At beginning, it describes the detailed deposition process of 35 nm InSb NCs film on SiO_2_/Si (111) substrate. Following the morphology, structures, optical, and electrical properties of the InSb NCs layer on SiO_2_/Si are presented. Finally, it provides that the NCs film has an average grain size of 12.3 nm, and the optical bandgap of InSb NCs film is about 0.26 eV.

### Experiment

InSb film has been deposited on SiO_2_/Si (111) substrate at room temperature by radio frequency (r.f.) magnetron sputtering using InSb alloy target. The native SiO_2 _layer on silicon, which was about 40 Å in thickness, was not removed prior to film growth. The base pressure is 10^-6 ^Pa to ensure a low level of impurities. The growing conditions are 55 W r.f. power, 0.6 Pa Ar pressure to ensure good crystallographic properties. The deposition experiments show that when a stoichiometric polycrystalline InSb target is used, the film is rich in Indium and the excess indium is presented on the surface of the film in the form of Indium balls. This has usually been explained by the relatively large equilibrium vapor pressure of group V metals in comparison with the group III metals. In this study with a purity of 99.95% antimony (Sb) and 99.995% of Indium (In), the InSb template was made by the water-cooled copper crucible in a vacuum arc furnace under Argon atmosphere. After confined, rod-like samples are suction-casted into Ф10 mm × 75 mm size. By using the wire cutting, the rod is cut into suitable shape specimens, cleaning with acetone, then is sealed into quartz tube under 10^-4 ^Torr vacuum degree, keeping for 24 h at 450°C to achieve uniformity of composition, following the composition of each specimen was determined by energy spectrum technique. The In/Sb ratio of the films was also measured, and after measuring more than 30 films, it was known that the film with atom ratio of 1:1 was grown by the target with the ratio of 1:0.82.

Post-deposition thermal annealing of film was accomplished in N_2 _atmosphere using rapid photothermal annealing system (RTA). The samples were annealed at 400°C for 2 min. The typical ramp-up and ramp-down temperature was approximately 25°C/s. The accuracy of temperature measurement was ± 2°C.

X-ray diffraction (XRD), transmission electron microscopy (TEM), and scanning electron microscopy (SEM) were used to study the crystallographic microstructure, the film thickness, and the surface morphology of the film, respectively. The powder XRD pattern was recorded on Philip X'Pert Discover (Cu Ka radiation, λ = 1.5406 Å), the scanning range is from 10° to 70°. Cross-sectional TEM image was recorded on JEOL JEM 2010F field emission electron microscope, using an accelerating voltage of 200 kV. The plan-scan images were taken on FEI Quanta 200 scanning electron microscope.

Fourier Transform infrared spectroscopy (FTIR) measurement was carried out by Perkin Elmer Lamda 2000 spectrum in absorption mode at room temperature in the spectral range between 2.5 and 25 μm, and the SiO_2_/Si wafer was used as the reference.

The *I*-*V *characteristics were recorded using a Keithley 6517A electrometer fitted with a voltage source. The field-effect phenomena were investigated on the XOI heterostructure.

## Results and discussion

**Figure 1:**
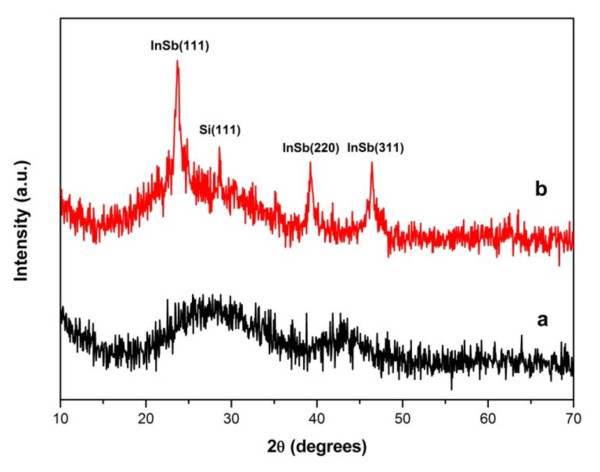
**X-ray patterns of InSb films: **(a) as-deposited and (b) RTA-annealed.

XRD patterns of both as-deposited InSb films and after RTA treatment at 400°C are shown in Figure 1. The XRD spectra show that the as-deposited InSb film is characterized by two humps indicating an amorphous structure in Figure 1a. After the film was annealed, three broad InSb diffraction peaks were observed, which were assigned to be (111), (220), and (311) orientations of InSb with a zinc-blend structure in Figure 1b. The full width at half maximum (FWHM) of each XRD peak was carefully measured, and the nanocrystals size was calculated by Scherr formula [13]

(1) D=0.9λ∕Bcosθ

where *λ *is the wavelength of the X-ray radiation (*λ *= 0.154 nm), *B *is FWHM of the peak (in radians) corrected for instrumental broadening, *θ *is Bragg angle, and *D *is the crystallite size. The InSb NCs size was calculated by this formula and the result is 12.3 nm.

**Figure 2:**
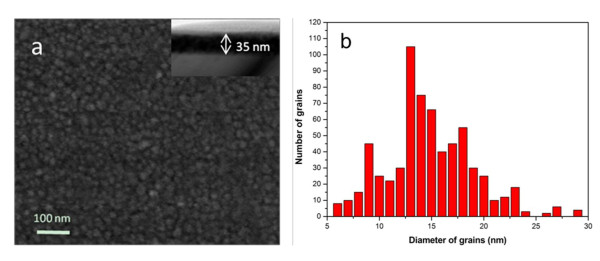
**SEM image and histogram of grains size distribution of InSb films: (a) **SEM image of the InSb NCs film. Inset shows the cross-sectional TEM image of InSb NCs film. **(b) **Histogram of the InSb NCs grains size distribution.

The InSb NCs' ultrathin film is shown in Figure 2a, it can be seen that the film has a uniformly deposited surface, composed of tightly packed spherical grains. The grains' size distribution was obtained and is shown in Figure 2b. The average grain size was found to be 14.6 nm. This value matches well with what we have obtained from the XRD measurements (12.3 nm). Good coverage and continuity of the film on the substrate indicates that the film could be used easily in various devices. A cross-sectional bright-field transmission electron micrograph of InSb NCs film is shown in the inset of Figure 2a, the thickness of the deposited InSb layer is about 35 nm.

**Figure 3:**
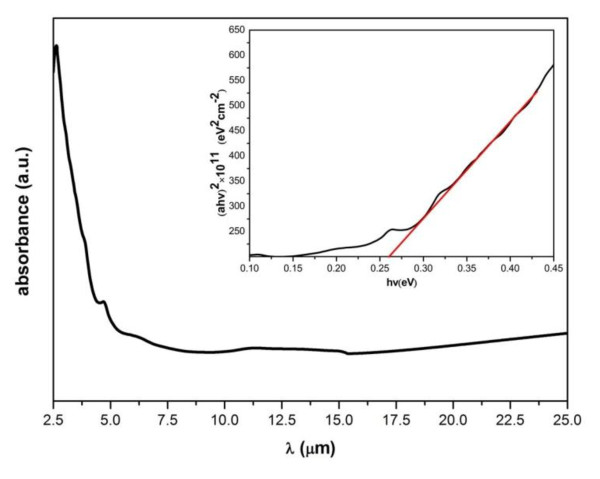
**FTIR image of the InSb NCs film**. Inset shows (α*hν*)^2 ^versus *hν *plots for the film.

Figure 3 shows that the measurement of infrared absorbance using FTIR in the wavelength range 2.5-25 μm. A sharp rise in absorbance is near 4.7 μm, which corresponds to the bandgap energy of the material (0.264). The typical bandgap energy for bulk InSb is 0.18 eV. Here, because of the quantum confinement effect exerted by the NC particles [14], a blue shift in the bandgap energy about 0.084 eV was observed. The optical bandgap of the film is determined by applying the Tauc model [15] in the high absorbance region,

(2) $$\alpha h\nu =D{\left(h\nu -E_{\text{g}}\right)}^{n}$$

where *hν *is the photon energy, *E*_g _is the optical bandgap, and *D *is a constant; it can be found that *n *= 2 corresponds to the indirect transition-type semiconductor and *n *= 1/2 leads to the direct transition type one [16]. Inset shows (*αhν*)^2 ^versus *hν *plots for the film. Following the conventional method of extrapolating the linear part of the Tauc plot, the optical bandgap energy for direct transition for the InSb NCs film was obtained, which is about 0.263 eV and agrees well with the value obtained directly from the absorption spectrum.

**Figure 4:**
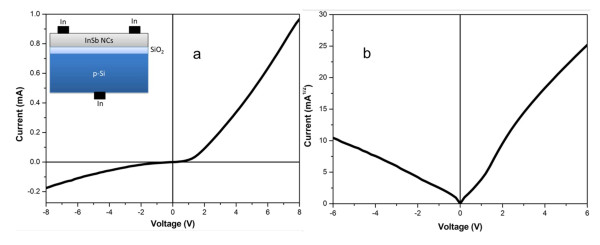
***I-V *characteristics: (a) ***I*-*V *characteristics of the InSb NCs/SiO_2_/p-Si heterojunction, inset shows the schematic diagram of the InSb NCs/SiO_2_/p-Si heterojunction, **(b) **the sqrt(*I*) plot of *I*-*V*.

Current-voltage characteristics of the XOI structure were measured and the results are presented in Figure 4. A schematic diagram of the device is shown in the inset in Figure 4a, The *I*-*V *characteristic is linear between two indium contacts on InSb NCs, indicating formation of ohmic contacts by indium. The *I*-*V *characteristics of the InSb NCs/SiO_2_/p-Si heterojunction in Figure 4a show a rectifying behavior and the turn-on voltage value is 1 V. The *I*-*V *characteristics are not linear and symmetric, meaning that the film does not make an ohmic contact to the Si substrate across the SiO_2 _layer. Figure 4b shows the transversal sqrt(*I*) versus *V *plot, suggesting a space charge limited current through the insulator layer.

## Conclusions

In summary, InSb film was grown on SiO_2_/Si (111) substrate according to r.f. magnetron sputtering and solid-phase recrystallization by using rapid thermal annealing. The detailed deposition process of InSb NCs film on SiO_2_/Si (111) substrate was described. The average grain size of InSb NCs film is about 12.3 nm. A sharp rise in absorbance was found to take place near 4.7 μm, and the optical bandgap of the InSb NCs film is about 0.26 eV. Compared with the bulk InSb, a blue shift in the bandgap energy (0.084 eV) was observed obviously. Regarding to the *I*-*V *characteristics of the InSb NCs/SiO_2_/p-Si heterojunction, it was found that the rectifying behavior and the turn-on voltage value is near 1 V.

## Abbreviations

FTIR: Fourier Transform infrared spectroscopy; FWHM: full width at half maximum; InSb: indium antimonide; *I*-*V*: current-voltage; NCs: nanocrystals; r.f.: radio frequency; RTA: rapid photothermal annealing system; SEM: scanning electron microscopy; SOI: silicon-on-insulator; TEM: transmission electron microscopy; XOI: semiconductor-on-insulator; XRD: X-ray diffraction.

## Competing interests

The authors declare that they have no competing interests.

## Authors' contributions

All the authors contributed equally to the work of this article. All authors read and approved the final manuscript.
